# Risk Prediction of Cerebral Infarction after Anterior Circulation Aneurysm Rupture in an Under-Equipped Centre

**DOI:** 10.21315/mjms2022.29.2.5

**Published:** 2022-04-21

**Authors:** Vayara Perumall VINODH, Abdul Rahman Izaini GHANI, Regunath KANDASAMY, Pulivendhan SELLAMUTHU, Mohd Sofan ZENIAN, Thamron KEOWMANI

**Affiliations:** 1 Department of Neurosciences, School of Medical Sciences, Universiti Sains Malaysia, Kelantan, Malaysia; 2 Department of Neurosurgery, Queen Elizabeth 2 Hospital, Sabah, Malaysia; 3 Clinical Research Centre, Queen Elizabeth Hospital, Sabah, Malaysia

**Keywords:** intracranial aneurysm rupture, subarachnoid haemorrhage, cerebral infarct, predictive model, VINODH score

## Abstract

**Background:**

Morbidity and mortality is high among aneurysm rupture patients. Despite surviving the initial rupture, morbidity is high as they suffer from vasospasm and cerebral infarction (CI). Most prediction tools for CI after aneurysmal subarachnoid haemorrhage (SAH) are complex and are not routinely available in all neurosurgical centres. Current therapies for prevention of CI are still debatable and selective usage among high-risk patients is advised. These factors necessitate a simple prediction model for identifying patients in the high risk group to initiate early preventive treatment of CI.

**Methods:**

Patients with anterior circulation aneurysm rupture who underwent surgical clipping were included. Demographic data and factors related to CI were collected to determine significance and were used to develop VINODH score (VS).

**Results:**

Two hundred patients were included with a median age of 51 years old. Multivariate analysis proved only four predictors were significant (*P* < 0.01) for developing CI. These predictors were used for the development of VS which was named after the main author and the model’s sensitivity was 79.0% and specificity was 83.0%. This highly predictive score (receiver operating characteristic [ROC]: 0.902) was internally validated.

**Conclusion:**

VS is a reliable tool for early identification of patients at risk of CI after aneurysmal SAH.

## Introduction

Intracranial aneurysms are not a rare diagnosis due to diagnostic breakthroughs. It mainly occurs in the anterior circulation of about more than 80%. The site of the origin is usually at the diversion of blood flow from the main arterial vessel causing some flow to continue to hit at the original trajectory instead of the diversion. Aneurysm rupture patients who died before reaching the hospital is about 10%. Despite survival, morbidity is high as they suffer from vasospasm and cerebral infarction (CI) (1).

Aneurysm ruptures are regarded as an emergency condition because re-rupture can happen and carries a mortality risk of about 70%. The risk of re-rupture is about 3%–4% within 24 h and 1%–2% each day within 30 days. Prevention of re-rupture can only be achieved by either surgical clipping or an interventional radiological approach. The most common cause of CI is either directly due to surgical complications or vasospasm. Vasospasm is preventable and early identification is crucial for medical intervention such as improving blood rheology and increasing blood pressure (2).

Brain aneurysm ruptures happens one in every 18 min in which up to one-third of the survivors have cerebral vasospasm. Vasospasm causes hypo-perfusion, which leads to CI if left untreated. Besides that, subarachnoid haemorrhage (SAH) also attenuates the effect of vasospasm. Therefore, early recognition is crucial for prevention (3).

A study using the data from the Prospective Registry of Subarachnoid Aneurysms Treatment (PRESAT) cohort identifies predictors for CI after aneurysmal SAH. PRESAT cohort was a retrospective look at aneurysm rupture patients’ management among clipping and coiling. It was a propensity score that showed that ischaemic morbidity was higher in the clipping group but did not specify if it was due to vasospasm from SAH (4). The aim of creating this new predictive score was mainly to identify high risk patients who are at increased risk of developing CI rather than just focusing on morbidity and mortality.

The currently available scores along with their outcomes and disadvantages are described in Table 1.

Besides the complexity and disadvantages of the currently available scores for the prediction of CI, the lack of resources in some centres also prevented the early identification of high risk patients for developing CI after SAH. These prompted a need for a new and simple prediction model that could be implemented in most centres. Thus, we decided to develop a score that will predict CI in all anterior circulation aneurysmal rupture patients with our own set of locally available parameters using non-invasive methods. In the case whereby this score is reliable, it can lead to the testing of this score in the posterior circulation aneurysm rupture patients with SAH.

The main objective is to determine significant predictors and develop a score to predict CI among anterior circulation aneurysmal SAH patients in underequipped neurosurgical centres. This score was tested to analyse the association with the patient outcome at discharge. The endpoint is to establish a high-risk target group patient for early preventive treatment of CI.

## Methods

### Research Design

This study aims to identify predictors that cause CI after aneurysmal SAH in patients who are surgically treated by aneurysm clipping.

### Research Location and Duration

This single centre study was conducted in Queen Elizabeth 2 Hospital, Sabah with a lack of modern invasive facilities such as digital subtraction angiography (DSA) and routine intracranial pressure (ICP) monitoring devices. The inadequacy in resources has forced us to only treat anterior circulation aneurysm patients at our hospital and complex posterior circulation aneurysm rupture patients are transferred to tertiary centres in the capital state of Malaysia for interventional or surgical procedures. Data was collected from 1st July 2014 to 31st January 2018.

### Study Population

All anterior circulation aneurysm rupture patients aged 18 years old and above with SAH who were admitted within 72 h from the onset of rupture, surgically treated by clipping and are at risk of developing cerebral infarction were included. The first 200 patients that fulfil all criteria retrospectively were included with the distribution of 100 cases and another 100 controls. These patients are enough to fit a model with up to 9 predictors if the rule of 10 is followed which was developed by Peduzzi et al. (5) in the *Journal of Clinical Epidemiolog*y stated that at a minimum of 10 events of the least frequent outcome should be observed per parameter. Missing data were handled by a complete-case analysis method.

The inclusion criteria are to have at least one repeated computed tomography (CT) scan at 24 h or when indicated in cases with new neurologic deficits or a prolonged state of impaired consciousness after surgical clipping of ruptured aneurysm. Cerebrospinal fluid (CSF) diversion was performed if the CT findings show acute hydrocephalus in which the size of both temporal horns should be more than 2 mm in width and the ratio of the largest width of frontal horns over internal diameter from inner-table to inner-table at this level is more than 0.5 and Evans’ index is more than 0.3 (10).

The exclusion criteria are all posterior circulation aneurysm, undetermined cause of SAH, absence of follow-up CT after surgery, unsure of pre-existing neurological deficit before the aneurysm rupture, patients who refused surgical clipping and presence of CI as a complication of surgery by improper clip placement or other indirect vascular injury.

### Method of Research

In this retrospective study, the analysed parameters were age, gender, the WFNS score, Fisher’s exact test grade, presence of hydrocephalus requiring drainage, presence of intracerebral haemorrhage, presence of intraventricular haemorrhage, Glasgow Coma Scale (GCS) and time of aneurysm rupture. Radiological parameters are reported by two radiologists while other parameters used in the score are reported by two neurosurgeons. The radiologists and the neurosurgeons have relevant experience of more than 3 years and were blinded to avoid inter-observer bias.

The definition of CI is taken as an area of new hypodensity seen in the CT scan within 21 days after aneurysm rupture. If the cause of hypodensity is doubtful, the case will be excluded.

In our centre, all patients with confirmed SAH on plain CT scan will get an immediate CT angiography (CTA). The CTA is only delayed if the patient has life-threatening hydrocephalus that requires urgent CSF diversion or in cases with deranged renal profile whereby prophylactic renal protective measures will be taken prior to CTA. Patients are then admitted to the neurosurgical high dependency unit. Nimodipine of 60 mg every 4 hourly and prophylactic levetiracetam of 500 mg every 12 hourly are given orally or via a nasogastric tube. Nimodopine is continued for 21 days from the onset of ictus while prophylactic levetiracetam is given for one week or continued regularly if the patient fitted.

All patients without severe life-threatening cardiac or renal problems will get maintenance of normal saline of 0.9% intravenous fluid of 4 mL/kg/day. Hydrocephalus is treated with an external ventricular or lumbar drain. All anterior circulation aneurysm rupture patients will be subjected to surgical clipping within 24 h from admission. Direct intraoperative papaverine of 60 mg is placed into the surgical field. Once clipped, hypertension is preferred aiming at the mean arterial pressure (MAP) of 90 mmHg–100 mmHg but if vasospasm is suspected intraoperative or at any time after clipping, the desired MAP is instead aimed at 100 mmHg–110 mmHg. Haematocrit and partial pressure of carbon dioxide (PaCO_2_) is maintained at 30%–35% and 35 mmHg–40mmHg, respectively.

### Statistical Analysis

The data were analysed using SPSS version 20 (IBM Corporation). Univariate analysis of the association between each predictor and cerebral infarct was performed using Fisher’s exact test. A binary logistic regression model was employed to build the prediction model for cerebral infarct. Backward selection with a *P*-value for removal of 0.05 was used to select the predictors into the main effects model. Interactions among the selected predictors were checked by adding one interaction term at a time to the main effects model and the Wald test was used to determine the significance at 0.05 level.

The overall fit of the resulting preliminary final model was assessed using Hosmer-Lemeshow goodness of fit (Ĉ), Pearson Chi-square (*χ*^2^) and deviance (D) statistics. The abilities of the model to predict and discriminate between the presence and absence of cerebral infarct were assessed using a classification table with a probability cut point of 0.5 and a plot of an area under the receiver operating characteristic (ROC) curve, respectively. Influential outliers were identified by plotting the Pregibon Delta-Beta influence statistic (Cook’s distance) against predicted probability. A covariate pattern with Cook’s distance greater than 1.0 was considered an influential outlier. The decision to keep or delete influential outliers was based on the change in the signs of the coefficients as well as the magnitude of change. In our case, no change in the signs was observed while the largest change was less than 20%, therefore, all observations were kept in the model. The resulting model after the last step was called the final model.

VINODH score (VS), which is named after the primary author was derived from the odds ratios (OR) in the final model. The utility of the score to predict CI was assessed by regressing using a binary logistic regression method. The model’s fit and diagnostics were checked. In addition, the observed and predicted probabilities were plotted together against the score to visualise the fit. The score’s utility to predict poor modified Rankin Scale (mRS) at discharge was assessed in a similar manner.

## Results

A total of 200 patients which comprised 100 events and 100 non-events were included. In this case, an event refers to aneurysm rupture patients who developed CI. There were more females with 59.5% compared to males. Majority of the patients presented with a good WFNS score and a poor Fisher’s exact test grade. Detailed demographic data and univariate analysis of the parameters are shown in Table 2. The median age of the patients analysed is 51. Missing data were handled by a complete-case analysis method.

### Identifying Significant Predictors and Developing VINODH Score

All nine predictors were then applied into a binary logistic regression model and backward selection with a *P*-value for removal of 0.05 was used to select the predictors for inclusion in the main effects model. As seen in Table 3; only female gender, CSF diversion for hydrocephalus, poor Fisher’s exact test grade and poor WFNS score proved as significant predictors for CI development which were used for the development of VS. As for the indicator, the VS was calculated by dividing the largest OR by the smallest OR and rounded to the nearest integer. The score of zero is applied as a reference score. Finally, VS was developed with a minimum score of 0 and a maximum score of 10.

**Figure 1:**
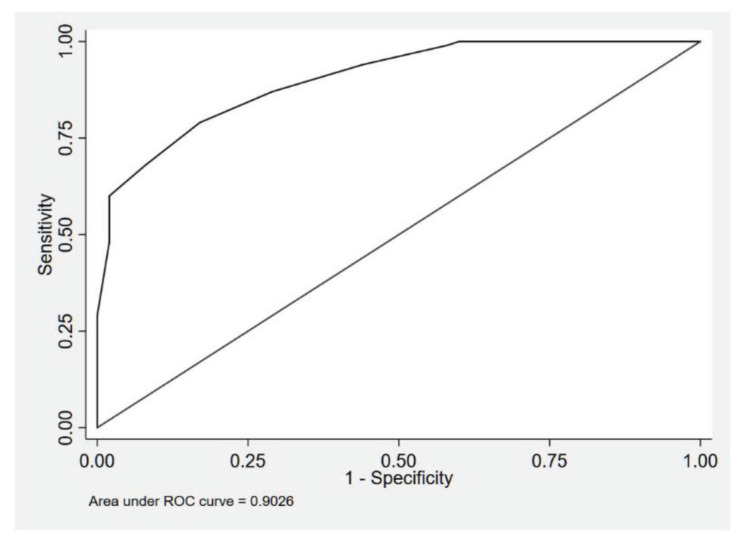
Area under the ROC curve for model of VS as the sole predictor of cerebral infarct (*n* = 200)

Univariate binary logistic regression using VS as the sole predictor of cerebral infarct showed high significance with a *P*-value of < 0.001 (OR 2.0). The observed probabilities for developing CI by VS is shown in Table 4 and is a form of internal validation of this score. The area under the ROC curve was 0.90 indicates outstanding discrimination (Figure 1). The addition of the WFNS and Fisher’s exact test grades has increased the sensitivity and specificity of the score. This ROC curve was only 0.83 for our data without including WFNS and Fisher’s exact test grades, which was better than using WFNS (0.77) or Fisher’s exact test (0.72) grades alone. Using a probability cut point of 0.5, the sensitivity of the model was 79.0% (true positive rate) and 83.0% specificity (true negative rate).

Patients were then stratified into risk groups of very low, low, moderate and high risk for developing CI based on the score’s occurrence of CI as seen in Table 4. This new score has a ‘positive predictive value’ (PPV) of 82% and a ‘negative predictive value’ (NPV) of 80%.

**Figure 2:**
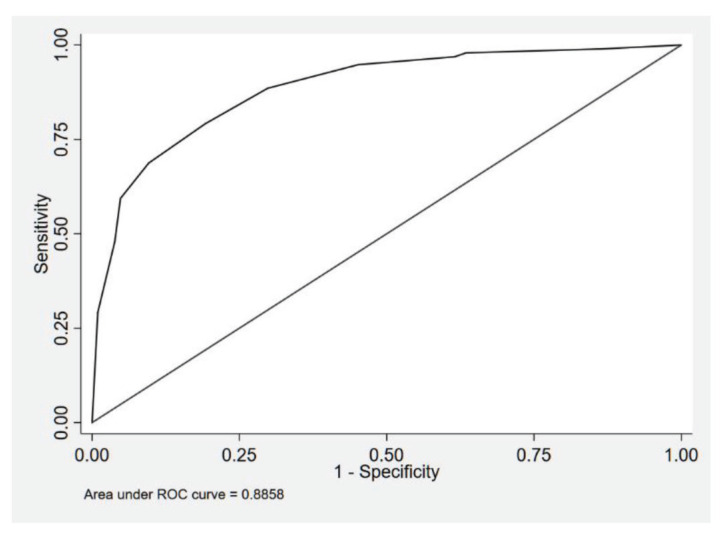
Area under the ROC curve for model of VS as the sole predictor of mRS score at discharge as the outcome (*n* = 200)

As for the analysis of the secondary outcome to correlate the mRS at discharge and VS, this study showed high significance with a *P*-value of < 0.001. The area under ROC curve was 0.89 indicating outstanding discrimination (Figure 2).

## Discussion

There are many available scores for CI prediction after aneurysmal SAH. Although poor Hunt & Hess score, poor WFNS score and poor Fisher’s exact test grades are associated with a bad outcome, CI after SAH is not always caused by vasospasm. It often occurs as a result of a multifactorial cascade. There are many independent predictors to be analysed. This score, despite being an easy and non-invasive predictive tool, is accurate in identifying high risk patients and helps to aim at their preventive management. It is proven to be more sensitive than using Fisher’s exact test grade (predictor of vasospasm) or WFNS score (predictor of mortality) alone.

The cohort from PRESAT study proves that multiple factors including vasospasm causes CI and reflects poor outcome among patients. These predictors were increasing age, admission WFNS grades IV to V, aneurysm re-rupture prior to admission, vasospasm induced CI, hydrocephalus requiring shunt, seizure, post-clipping haemorrhagic complication, pneumonia, sepsis and post-coiling ischaemic complication. Symptomatic vasospasm was proven medically treatable, hence it warrants prompt recognition and prevention (4).

CI after aneurysmal SAH can be divided into early or delayed in which delayed usually occurs after 21 days of an initial rupture. Surgical clipping is a risk for early CI and angiographic vasospasm aids in the development of delayed CI. Early CI causes poorer outcomes compared to delayed CI (6).

Re-rupture and vasospasm contributed to the chief causes of morbidity and mortality among aneurysm rupture survivors. Nimodipine, a dihydropyridine calcium channel blocker, acts on the vascular smooth musculature by inhibiting calcium entry and stops calcium-induced constriction. It has greater effects on cerebral vasculature and has been able to significantly reduce delayed CI (7).

Vasospasm is usually divided into clinical and radiological in which radiological is best diagnosed on DSA. Clinical vasospasm is regarded as a drop in consciousness level preceding a focal deficit and usually occurs after exclusion of other causes such as re-rupture, hydrocephalus, metabolic issues or surgical problems. The onset is usually insidious and occurs four days to 9 days after the first rupture (8).

CI is a known sequel of aneurysmal SAH despite treatment mainly due to vasospasm but this condition is inadequately understood. The cause of CI is multifactorial such as hydrocephalus, thick cisternal clot, superfluous manipulation of vessels during surgery and aneurysm re-rupture prior to clipping. Thus, it is indicated for a study to identify patients that are at risk of developing CI after aneurysm rupture (9).

A study shows that CI is frequent in patients with angiographic vasospasm, which is up to 43% but patients also develop CI without the presence of vasospasm. This number can be misleading for clinicians to assume that CI is mostly dependent on vasospasm while there are other mechanisms and factors which contribute to the development of CI after aneurysm rupture (10). CI also depends on the presence of deeper subcortical ischaemic foci adjacent to the rupture site (11). Transcranial doppler (TCD) and angiographic results often do not correlate and point to a mechanism other than the well-defined vasospasm at the level of the circle of Willis. Small vessel spasm and the idea of endothelial failure with narrowed lumens are usually found through histological examination (12–13). Failure in autoregulation also may lead to reduced cerebral perfusion causing CI (14).

Several theories have been suggested for the pathogenesis of CI after SAH. The accepted theories are narrowing of vessels causing hypoperfusion, disruption of the blood-brain barrier causing early cerebral injury and an autoregulatory problem with microthrombosis (15). Delayed CI is suggested as an occurrence due to inflammatory processes that lead to neuronal necrosis and apoptosis (16–17).

Prevention of CI is currently the mainstay of a treatment strategy. ‘Behaviour’ score was developed as an early risk score for CI based on the clinical parameters after aneurysmal SAH. This score, which showed the absolute risk for CI (area under curve = 0.806, *P* < 0.001) and prediction of poor clinical outcome at discharge (*P* < 0.0001); has high diagnostic accuracy in identifying patients at risk for developing CI after aneurysmal SAH (18). However, we were not able to apply this score to our daily neurosurgical practice. This is because not all neurosurgical centres use Hunt & Hess score in assessing patients as it can be very subjective to the clinician and not all centres also have a routine DSA facility or adequate ICP monitoring devices due to financial constraints.

We realised that currently available scoring systems were complex and require a higher financial burden or invasive techniques to be established whereby some neurosurgical centres will not be able to cope. This prompted the need for a new scoring system that will have the same outcome or better but with simpler and using non-invasive parameters. Neurosurgery is a rapidly growing field in most developing countries, especially where most of the funding is siphoned to core neurosurgical procedures. In our centre, the diagnosis of ruptured aneurysm is confirmed by performing a CTA. However, we do not have a DSA facility. Furthermore, we have limited ICP monitoring devices, which are mainly reserved for severe brain trauma cases and prevents routine use for most patients.

Out of the nine analysed parameters, only four parameters were included after multivariate analysis. Despite the idea that increasing age is still connected with increased morbidity; in our study, we found that there is no significance to the occurrence of CI if multifactorial compounding factors are applied. Aneurysm rupture rarely causes large ICH and if present, it is associated with higher mortality but in our study, we found that ICH is not a significant predictor of CI after SAH. The same goes with the presence of intraventricular haemorrhage (IVH) as it is not a significant predictor. We wanted to analyse if the time of aneurysm rupture predicts the occurrence of CI. This is because the common belief of delayed presentation to the hospital if the rupture occurs at night could prolong the time from rupture to treatment but, this also has been proved insignificant between day and night rupture.

The identification of high-risk patients for developing CI after aneurysmal SAH will require an early intervention aimed at preventive measures. There are also arguments and critics on the necessity of the ‘triple-H’ therapy, which is currently avoided in most centres because routine use of this therapy among all aneurysm rupture patients have been proven to be detrimental. ‘Triple-H’ therapy that comprises hypertension, hypervolemia and haemodilution is of standard practice in the past in most centres for vasospasm. Controversy still surrounds this therapy especially due to the complications of hypervolemia which can cause pulmonary oedema, hyponatremia, renal medullary washout and cerebral oedema. Identification of a high-risk group of patients will allow the use of this therapy selectively in particular with avoidance of hypervolemia (19–21).

Fluid therapy commonly used in the high-risk group of patients are crystalloids such as normal saline 0.9% intravenous fluid of 4 mL/kg/day to maintain hypervolemia state although no data to support hypervolemia over euvolemia. As for hypertension, vasopressors such as phenylephrine and noradrenaline are used with MAP aimed at about 100 mmHg–120 mmHg (22). Haemodilution also lacks data to support its use, but the proposed mechanism is that a decreased haematocrit improves blood rheology and increases flow through the vasospastic vessel. The common agents used are intravenous albumin and fresh frozen plasma. Haemodilution with these agents are usually avoided at the initial stages as hypervolemia itself causes dilution and these agents are associated with hypersensitivity reactions (23–24).

The advantage of magnesium in SAH stems from its biochemical properties as a potent antagonist of calcium which is cost effective and relatively safe with minimal side effects. Serum magnesium is maintained at 1.0 mmol/L values for the first seven days after aneurysm rupture as there is a higher risk of infarct during this period. Currently, this is the emerging standard of practice in most centres among younger neurosurgeons. However, the role of magnesium in treating CI after aneurysmal SAH is still debated. A small study showed a trend towards lesser symptomatic vasospasm with magnesium but a large, controlled trial of continuous magnesium infusion did not find conclusive effects on CI. Magnesium also has no benefit for prophylaxis of CI in SAH patients (25–28).

About 70% of patients with aneurysmal SAH have radiological vasospasm and only about one-third of these patients showed clinical vasospasm symptoms. Most of the time, ischaemic injury has already occurred by the time of vasospasm detection and the onset of clinical symptoms may be sudden or insidious. Due to the difficulty in detection, especially among patients with impaired consciousness levels, it is important to closely monitor these patients. Initiating treatment to prevent irreversible neurological deficits and mortality can be achieved if high risk patients are identified early (29).

The limitations in this study were mainly the design of the retrospective data collection, which would not represent a prospective cohort in improving the accuracy of the created score. The selective sample of only anterior circulation aneurysm rupture patients is because we do not surgically treat complex posterior circulation aneurysm patients in our centre. This would not represent the whole population of aneurysm rupture patients in Sabah. Inclusion of these patients in the future study would improve the accuracy of this score. The current VS has only gone through internal validation with the pre-existing sample population and a future multicentre external validation is required.

## Conclusion

VS is a simple and reliable tool, which is accurate for early identification of patients at risk of CI after anterior circulation aneurysmal SAH and thus, can be used in identifying a target group of high-risk patients for early preventive treatment of CI.

## Figures and Tables

**Table 1:** Available scores along with their outcomes and disadvantages

Scores	Methodology	Outcome parameters	Advantages	Disadvantages
Behaviour score (18)	Retrospective analysis of 674 consecutive patients admitted with spontaneous SAH with a prospective validation of score.	Concise early risk score for predicting CI occurrence.	Identifying patients at risk for developing CI after aneurysmal SAH.	Complex as requires invasive monitoring technique and also fully equipped neurosurgical centre.
Hunt and Hess score (30)	Modification of Botterell’s classification applied retrospectively to 275 consecutive cases of intracranial aneurysm.	Vasospasm risk and survival.	Quantifies the risk vasospasm development and survival outcome in patients according to consciousness level and neurological deficit.	Grading does not include radiological criteria. Clinical grading of consciousness level is also subjective and might not be the same among different clinicians.
World Federation of Neurosurgical Societies (WFNS) score (31)	Retrospective analysis of 3,521 patients from 68 countries enrolled in the International Cooperative Aneurysm Study (ICAS).	Mortality and morbidity.	Patient outcome described based on grades and more objective for consciousness level based on GCS.	Grading does not include radiological criteria.
Fisher grading scale (32)	Prospective analysis of 47 patients with aneurysm who had CT within first 5 h after rupture and angiography during period of vasospasm.	Vasospasm risk.	Estimates the risk of development of vasospasm based on radiological grading.	Purely radiological classification and does not reflect other clinical parameters as the cause of CI is usually multifactorial.

**Table 2:** Demographic data and univariate analysis of each predictor against CI (*n* = 200)

Predictor	*n*	Cerebral infarct *n* (%)		*P*-value^a^

		No	Yes	
Age (years old)				< 0.001
Less than 51	97	62 (63.9)	35 (36.1)	
51 or more	103	38 (36.9)	65 (63.1)	
Hydrocephalus requiring CSF diversion				< 0.001
No	101	75 (74.3)	26 (25.7)	
Yes	99	25 (25.3)	74 (74.7)	
WFNS score				< 0.001
Good: 1–3	114	84 (73.7)	30 (26.3)	
Poor: 4–5	86	16 (18.6)	70 (81.4)	
Fisher’s exact test grade				< 0.001
Good: 1–2	64	54 (84.4)	10 (15.6)	
Poor: 3–4	136	46 (33.8)	90 (66.2)	
GCS				< 0.001
Good: 9–15	161	94 (58.4)	67 (41.6)	
Poor: 8 or less	39	6 (15.4)	33 (84.6)	
Intraventricular haemorrhage				< 0.001
No	132	79 (59.9)	53 (40.1)	
Yes	68	21 (30.9)	47 (69.1)	
Sex				0.084
Male	81	47 (58.0)	34 (42.0)	
Female	119	53 (44.5)	66 (55.5)	
Intracerebral haemorrhage				0.535
No	141	73 (51.8)	68 (48.2)	
Yes	59	27 (45.8)	32 (54.2)	
Aneurysm rupture time				0.887
Day: 06:00–17:59	106	54 (50.9)	52 (49.1)	
Night: 18:00–05:59	94	46 (48.9)	48 (51.1)	

Note:

a Fisher’s exact test

**Table 3:** Final binary logistic regression model and final model of VS (*n* = 200)

Predictor	OR	95% Confidence interval for OR	*P*-value a	VS
Fisher’s exact test grade				
b**Poor: 3–4**	**11.0**	3.9, 30.9	**< 0.001**	**3**
cGood: 1–2	1.0			0
Gender				
b**Female**	**3.5**	1.5, 8.4	**0.004**	**1**
cMale	1.0			0
Hydrocephalus requiring CSF diversion				
b**Yes**	**6.2**	2.7, 14.0	**< 0.001**	**2**
cNo	1.0			0
WFNS score				
b**Poor: 4–5**	**12.3**	5.2, 29.5	**< 0.001**	**4**
cGood: 1–3	1.0			0
Total score				10

Notes:

a Wald test;

b indicator;

c reference

**Table 4:** Observed risk and probabilities for CI by VS

VS	Risk group	Absence of CI [*n* (%)]	Presence of CI [*n* (%)]	95% Confidence interval for predicted CI
0	Very low	40 (100)	0 (0)	0.000–0.044
1				0.006–0.079

2	Low	31 (70.5)	13 (29.5)	0.027–0.139
3				0.078–0.234
4				0.180–0.367

5	Moderate	27 (50)	27 (50)	0.335–0.533
6				0.511–0.708
7				0.670–0.852

8	High	2 (3.2)	60 (96.8)	0.793–0.939
9				0.878–0.981
10				0.931–0.997
